# A Wireless Electro-Optic Platform for Multimodal Electrophysiology and Optogenetics in Freely Moving Rodents

**DOI:** 10.3389/fnins.2021.718478

**Published:** 2021-08-16

**Authors:** Guillaume Bilodeau, Gabriel Gagnon-Turcotte, Léonard L. Gagnon, Iason Keramidis, Igor Timofeev, Yves De Koninck, Christian Ethier, Benoit Gosselin

**Affiliations:** ^1^Smart Biomedical Microsystems Laboratory, Department of Electrical Engineering, Université Laval, Québec, QC, Canada; ^2^Department of Psychiatry and Neuroscience, CERVO Brain Research Centre, Université Laval, Québec, QC, Canada

**Keywords:** electrophysiology, optogenetics, photostimulation, freely moving, digital signal processing, wireless, implantable device

## Abstract

This paper presents the design and the utilization of a wireless electro-optic platform to perform simultaneous multimodal electrophysiological recordings and optogenetic stimulation in freely moving rodents. The developed system can capture neural action potentials (AP), local field potentials (LFP) and electromyography (EMG) signals with up to 32 channels in parallel while providing four optical stimulation channels. The platform is using commercial off-the-shelf components (COTS) and a low-power digital field-programmable gate array (FPGA), to perform digital signal processing to digitally separate in real time the AP, LFP and EMG while performing signal detection and compression for mitigating wireless bandwidth and power consumption limitations. The different signal modalities collected on the 32 channels are time-multiplexed into a single data stream to decrease power consumption and optimize resource utilization. The data reduction strategy is based on signal processing and real-time data compression. Digital filtering, signal detection, and wavelet data compression are used inside the platform to separate the different electrophysiological signal modalities, namely the local field potentials (1–500 Hz), EMG (30–500 Hz), and the action potentials (300–5,000 Hz) and perform data reduction before transmitting the data. The platform achieves a measured data reduction ratio of 7.77 (for a firing rate of 50 AP/second) and weights 4.7 g with a 100-mAh battery, an on/off switch and a protective plastic enclosure. To validate the performance of the platform, we measured distinct electrophysiology signals and performed optogenetics stimulation *in vivo* in freely moving rondents. We recorded AP and LFP signals with the platform using a 16-microelectrode array implanted in the primary motor cortex of a Long Evans rat, both in anesthetized and freely moving conditions. EMG responses to optogenetic Channelrhodopsin-2 induced activation of motor cortex *via* optical fiber were also recorded in freely moving rodents.

## 1. Introduction

New tools to study the brain microcircuits of laboratory animals are highly sought after to advance our knowledge of the brain physiology and pathology. The rapid development in optogenetics and electrophysiology have allowed a wide array of different experimental approaches to better understand neural circuits (Balasubramaniam et al., 2018). Optogenetics allows the control of specific genetically modified neurons by light, which improves neural stimulation selectivity and avoids interference with parallel electrophysiological recordings compared to electrical stimulation. Small wireless implantable platforms combining both optogenetic stimulation with electrophysiological recording capability have been designed to study the brain dynamics in small laboratory animals (Gagnon-Turcotte et al., 2017a, 2020; Mesri et al., 2018; Bilodeau et al., 2020). However, performing high-resolution electrophysiology recordings and optical stimulation in small freely moving animal without interfering with their natural behavior remains challenging (Buzsáki et al., 2015).

Assessing multiple electrophysiological signal modalities in parallel is a critical requirement in several experimental settings (Mehring et al., 2003; Jackson et al., 2006; Ethier and Miller, 2015; Watson et al., 2018). Simultaneous recording of LFP and AP signals is crucial in understanding their respective roles, relations, and complementarity (Watson et al., 2018). The information extracted from these two distinct signal modalities has shown to yield better prediction results during reach and grasp tasks in non-human primates (Mehring et al., 2003; Abbaspourazad et al., 2021). Moreover, the development of reliable brain machine interfaces often require to perform parallel recording and stimulation in the motor cortex or other brain structures, while monitoring the EMG signals in muscles (Jackson et al., 2006; Ethier and Miller, 2015). LFP and AP signals are typically recorded from the same electrode implanted in the central nervous system, while EMG is recorded from a separate electrode inserted in the muscle or located under the skin. The amplitudes and frequency bands of AP, LFP, and EMG, respectively range from 300 to 5,000 Hz and 50 to 500 μV (Liu et al., 2013), 1 to 500 Hz and 250 to 5,000 μV (Soltani et al., 2019), and 10 to 300 Hz and 50 to 500 μV (Soltani et al., 2019). Capturing all these waveforms concurrently requires a dedicated strategy. A minimum sampling rate of 10 kS/s is needed, while 25 kS/s is most desirable (Gagnon-Turcotte et al., 2017b), which can produce very high data rates that cannot be handled with low power telemetry in a system including multiple channels. Recording the full neural signal bandwidth on 32 channels in parallel, at 20 kS/s and on 16 bits yields a data rate above 10.24 Mb/s, which cannot be supported by low-power BLE transceivers. Data reduction and/or data compression strategies have proven essential to increase the channel count and resolution, while decreasing the size and the power consumption of such resources constrained devices.

Since the APs are discreet events with high frequency content, data reduction methods mainly focus on this specific modality (Wu and Tang, 2011; Liu et al., 2013; Gagnon-Turcotte et al., 2017b), but each modality has its specific characteristics requiring a custom data reduction scheme. For instance, AP data reduction often initiates by performing high-pass filtering and signal detection (Wu and Tang, 2011; Liu et al., 2013) using a fixed or a dynamic threshold which cannot be performed on continuous low-frequency signals such as LFP. Only the detected APs are transmitted in this scheme, which can greatly reduce the data rate. A combination of AP detection and data compression can yield compression ratios (CR) above 500 (Gagnon-Turcotte et al., 2017b). This type of method, known as *activity dependent* reduction method, can significantly decrease the amount of data, but requires the signal to be high-pass filtered to avoid any LFP contamination, thus, in this approach, the LFPs, which lies at low frequencies, must be separated from the APs right at the beginning.

Neural signal recording systems that rely on custom integrated circuits (IC), often use a sophisticated analog signal processing front-end including different types of analog filters to isolate the preferred signal band directly before digitization (Wu and Tang, 2011; Biederman and al., 2015; Kassiri et al., 2017; Lopez et al., 2017; Gagnon-Turcotte et al., 2018). These solutions can be really effective when recording within only one signal band, or when the data rate is not representing a bottle neck, as it is the case in tethered settings. A custom IC using one dedicated filter and one analog-to-digital converter (ADC) per signal modality has been described before (Liu et al., 2017). This solution, which aims at performing closed-loop control, is trading signal integrity for extracted features. The solutions presented in Perelman and Ginosar (2005) and Bihr and Ortmanns (2012) both use custom chips including two separation filters and 2 ADCs per channel to capture the two separate bands i.e., low- and high-frequency bands simultaneously. This is because the amplitude of the LFP signals can be 10 times higher than the amplitude of extracellularly recorded AP signals. The dynamic range can then be optimized *a posteriori* to reduce the number of bits needed for each modality. However, this approach is limited to only small data reduction factors (e.g., RD < 2 for APs as shown in Bihr and Ortmanns, 2012) at the cost of a lot of additional hardware. Additionally, custom chip solutions typically require longer development time compared to COTS solutions.

Wavelet compression has been applied to AP before in real-time (Schmale et al., 2013; Gagnon-Turcotte et al., 2018). The study presented in Schmale et al. (2013) provides a comparison to determine the best suited wavelet to process AP and LFP, but does not provide insight on how to utilize the results inside a miniaturized device. In contrast, Gagnon-Turcotte et al. (2018) has demonstrated a fully integrated wavelet detection and compression core embedded inside a 10-channel electrophysiology chip for recording AP and LFP separately, but this system cannot capture AP and LFP simultaneously, and was not validated in freely moving rodents.

Here, we present the design of a wireless electro-optic platform providing multimodal electrophysiology recording from 32 channels as well as optogenetic stimulation, and its validation in freely moving laboratory animals. The presented platform, which is based on commercial off-the-shelf components, can (i) extract AP, LFP and/or EMG signals concurrently, over 32 channels, (ii) perform optogenetics stimulation over four channels along with the electrophysiology recording, and (iii) be utilized in freely moving rodents without any hardwired connection. The system includes a real time digital signal processing core running on a low-power FPGA embedded in the platform to detect and separate the different signal modalities on the fly, and a data compression core to increase the resolution. We validated the system *in vivo* in both anesthetized and freely moving rodents and showed that the system can separate different types of electrophysiological waveforms simultaneously while compressing and transmitting the data wirelessly and in real time.

## 2. Materials and Methods

### 2.1. System Overview

**Figure 1:**
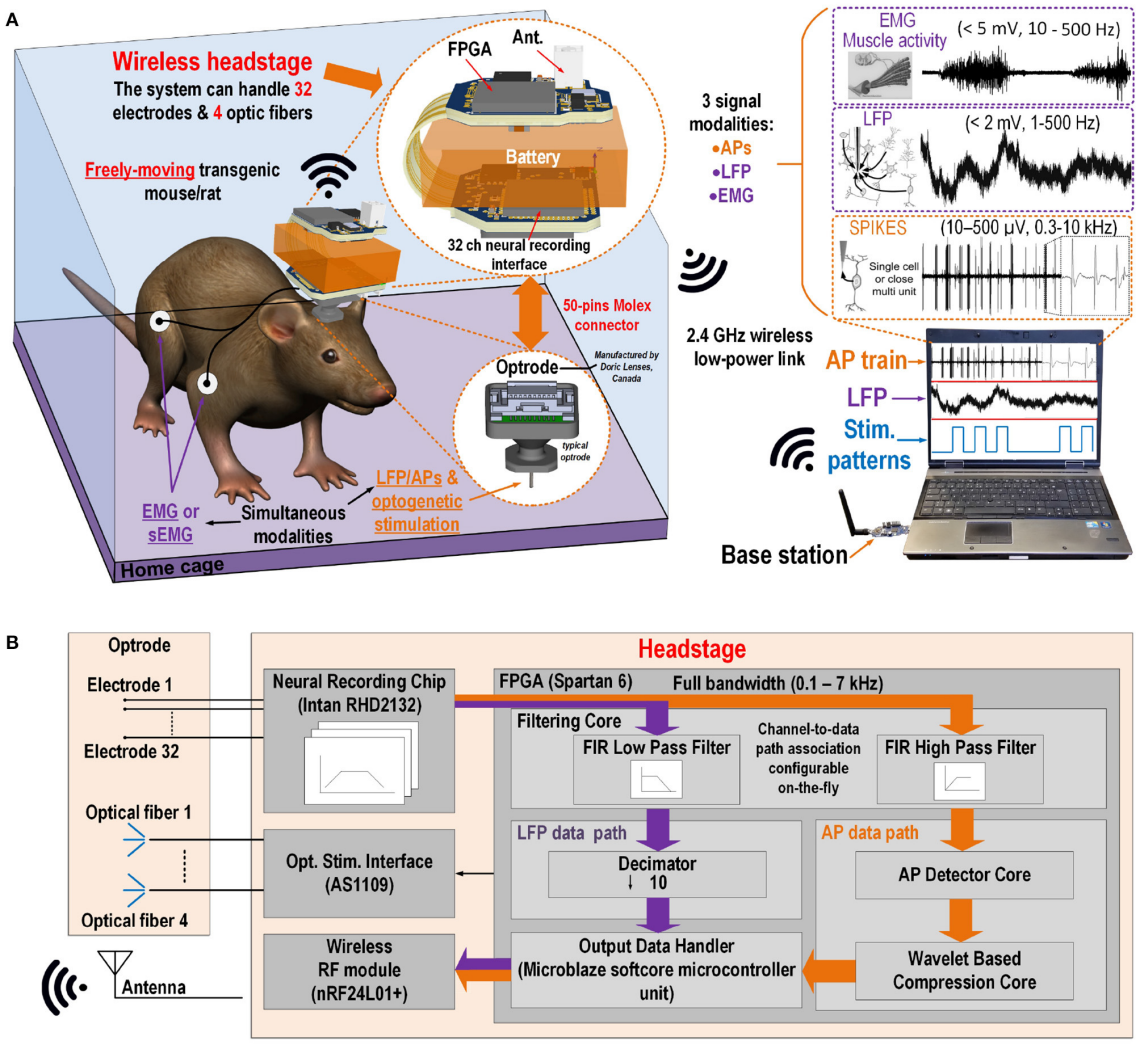
**(A)** On the left, the concept of the platform connected on an optrode secured on the head of a rodent and to EMG electrodes going to its arm. On the right, the base station with the computer host communicating with the platform. **(B)** System level concept of the platform.

The concept of the proposed wireless electro-optic platform is shown in Figure 1A. A detachable platform is connected to an implantable interface, i.e., an optrode or a microelectrode array (Figure 1B), which is secured on the head of a laboratory animal. It can record different types of electrophysiology signals using up to 32 microelectrodes in parallel at 20 ksps (16-bits per samples), and perform optical stimulation using four fiber-coupled LEDs. The presented platform improves the system described in Gagnon-Turcotte et al. (2017b) with a new signal processing core to capture simultaneously multiple electrophysiology signal modalities and a new light-weight format. Additionally, the platform was tested with freely moving rodents. It includes a robust digital signal processing strategy to capture and separate the electrophysiological signal modalities, i.e., LFP/EMG and AP on the fly. Separating these signals in multiple paths inside the system allows to decrease the data rate and the power consumption by performing signal detection and wavelet compression inside the low-power embedded FPGA. Using multiple data paths to separate both LFP and AP inside the platform is the key to perform AP detection and data compression in this design. The system includes a new strategy to extract three signal modalities (i.e., AP, LFP, and EMG) in parallel within the same system. The platform benefits from an optimized integration strategy leveraging a lightweight printed circuit board and a low-power FPGA to implement the custom digital signal processing cores.

The platform encompasses four main building blocks implemented on a rigid-flex printed circuit board (PCB) including (1) a RHD2132 neural recording interface from *Intan Technologies*, USA, (2) a AS1109 4-channel precise LED driver for optical stimulation from *AMS*, Austria, (3) a nRF24l01p 2 Mbps (on-air) wireless transceiver from *Nordic Semiconductor*, Norway, and (4) a small-sized Spartan-6 (XC6LSX16, 8 × 8 mm^2^ 196-CSPBGA package) low-power field-programmable gate array (FPGA) from *Xilinx*, USA.

#### 2.1.1. Neural Recording Interface

The RHD2132 from *Intan Technologies* provides the platform with a 32-channel neural recording interface which can collect differential signal from 32 electrodes and a common reference, with a maximum amplitude of −5 to 5 mV and a sampling rate of up to 30 ksps. The low-pass cutoff frequency of the bandpass filter is configurable from 100 Hz to 20 kHz, while the high-pass is configurable from 0.1 to 500 Hz, which allows targeting a wide range of electrophysiology signals. It should be noted that every channel reuses the same bandpass filter parameters. The chip uses a small 56 pins QFN package, the size of which is only 8 × 8 mm^2^.

#### 2.1.2. Optical Stimulation

The optical stimulation module uses the AS1109 LED driver circuit from *AMS, Austria* which can drive up to 8 LEDs. Each output channel of the chip can supply 0.5–100 mA with a 8-bits resolution. Each LED is driven by two driver output channels in parallel to provide a maximum current of 200 mA to each LED. The system can use 8 LEDs, like the LB G6SP from OSRAM Opto Semiconductors, which can deliver 250 mW/mm^2^ at the output of the optical fiber (Gagnon-Turcotte et al., 2017b) when a driving current of 150 mA is provided by the LED driver, for triggering neuronal activity in transgenic animals over-expressing an opsin (LeChasseur et al., 2011). The AS1109 LED driver uses a pins QFN package, the size of which is only 4 × 4 mm.

#### 2.1.3. Wireless Transceiver

The platform uses a nRF24l01p wireless transceiver chip from *Nordic Semiconductor*. This transceiver uses a center frequency of 2.45 GHz in the corresponding Industrial, scientific and medical frequency band and a custom protocol by *Nordic Semiconductor*. It provides a maximum data rate of 2 Mbps in the air, a 11.3 mA current in data transmitting mode and 13.5 mA in receiving mode at 2 Mbps. This wireless transceiver chip allows flexibility with respect to the communication protocol and provides a high effective data rate of up to 1.4 Mbps (Gagnon-Turcotte et al., 2017b) for a low-power consumption. This chip uses a 20 pins QFN package, the size of which is only 4 × 4 mm.

#### 2.1.4. Digital Signal Processing Cores

In this design, a the XC6SLX16 Spartan 6 FPGA from *Xilinx Inc, USA* is utilized to perform signal processing inside the platform in real-time. A custom DSP core module developed and described previously by our group (Gagnon-Turcotte et al., 2017b; Bilodeau et al., 2020) is used to perform signal separation of the LFP, EMG, and AP and AP detection and wavelet signal compression. The FPGA controls all the custom digital cores as well as other modules and chips, i.e., the neural interface, the wireless transceiver and the optical stimulation, using a *Microblaze* microcontroller (MCU) softcore. The block diagram of the system along with the internal modules implemented inside the FPGA are depicted in Figure 1B.

#### 2.1.5. PCB System Design

**Figure 2:**
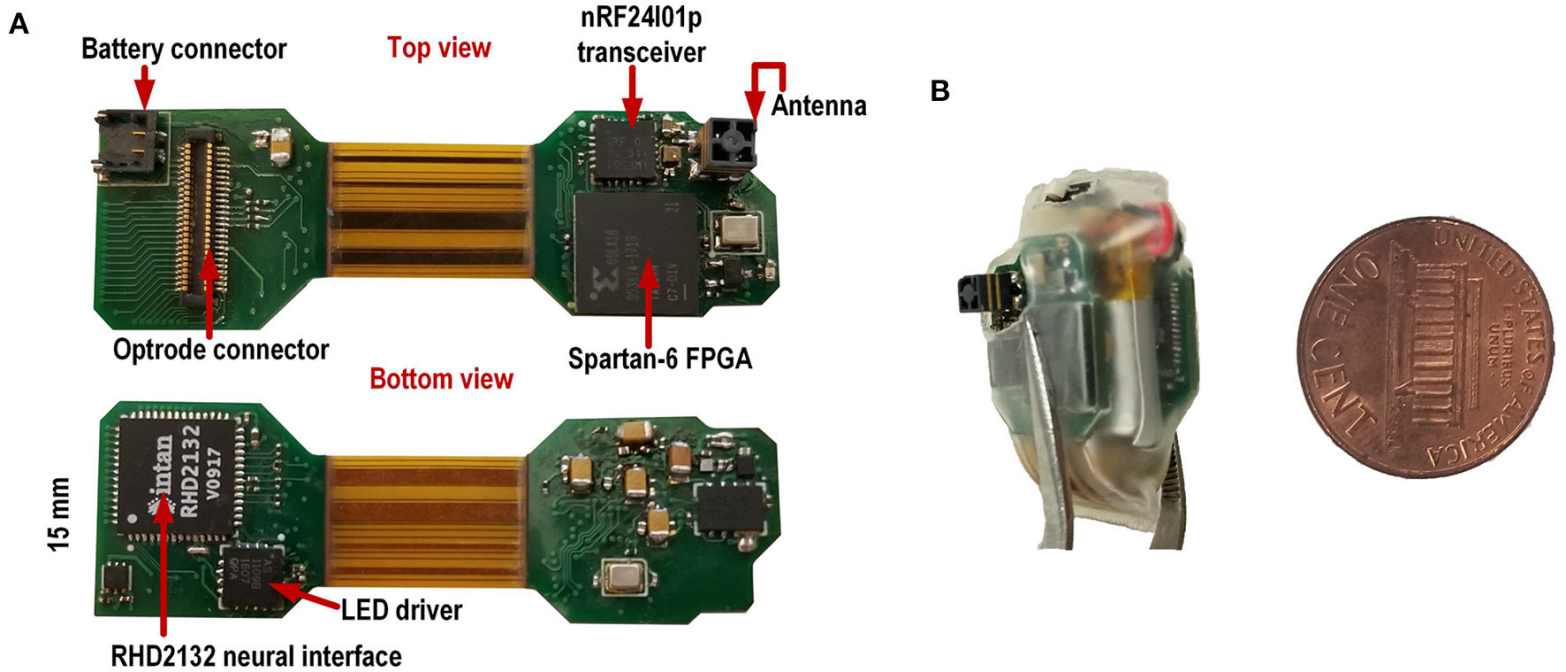
**(A)** Two-sided view of the rigid-flex PCB when not folded. **(B)** System in its plastic enclosure.

The platform was implemented on a thin rigid-flex printed circuit board as shown in Figure 2A. Since the system is designed for enabling experiments in small rodents, it has a low weight (<3.5 g; Gagnon-Turcotte et al., 2017a) and provides sufficient autonomy to accommodate different types of behavioral experiments (30–60 min). All components are mounted on a rigid-flex PCB including two rigid sections and a flexible section. The top rigid section holds the low-power transceiver and the FPGA, while the bottom rigid section holds the optical stimulator components (i.e., the LED driver), the neural recording interface and a Molex connector (#0559090574) for connecting the system to an optrode (see Figure 1B). The rigid sections are connected to each other using flexible sections. The system is folded around the battery to achieve a very compact size (Figure 2B). The final weight of the system is 4.68 g including a 100 mAh battery (Model 051417, MYD Technology), a plastic packaging and an on/off switch. The electronic system represents only 36% of the total weight (~ 1.7 g), thus lower final weight can be achieved by reducing the battery size and capacity and/or by removing the protective plastic. This specific packaging and battery size are suited for experimental settings involving live laboratory mice or rats.

### 2.2. Multimodal Extraction Strategy

Our signal extraction strategy allows collecting AP, LFP, and EMG signals in parallel using all or a subset of the 32 channels available.

**Figure 3:**
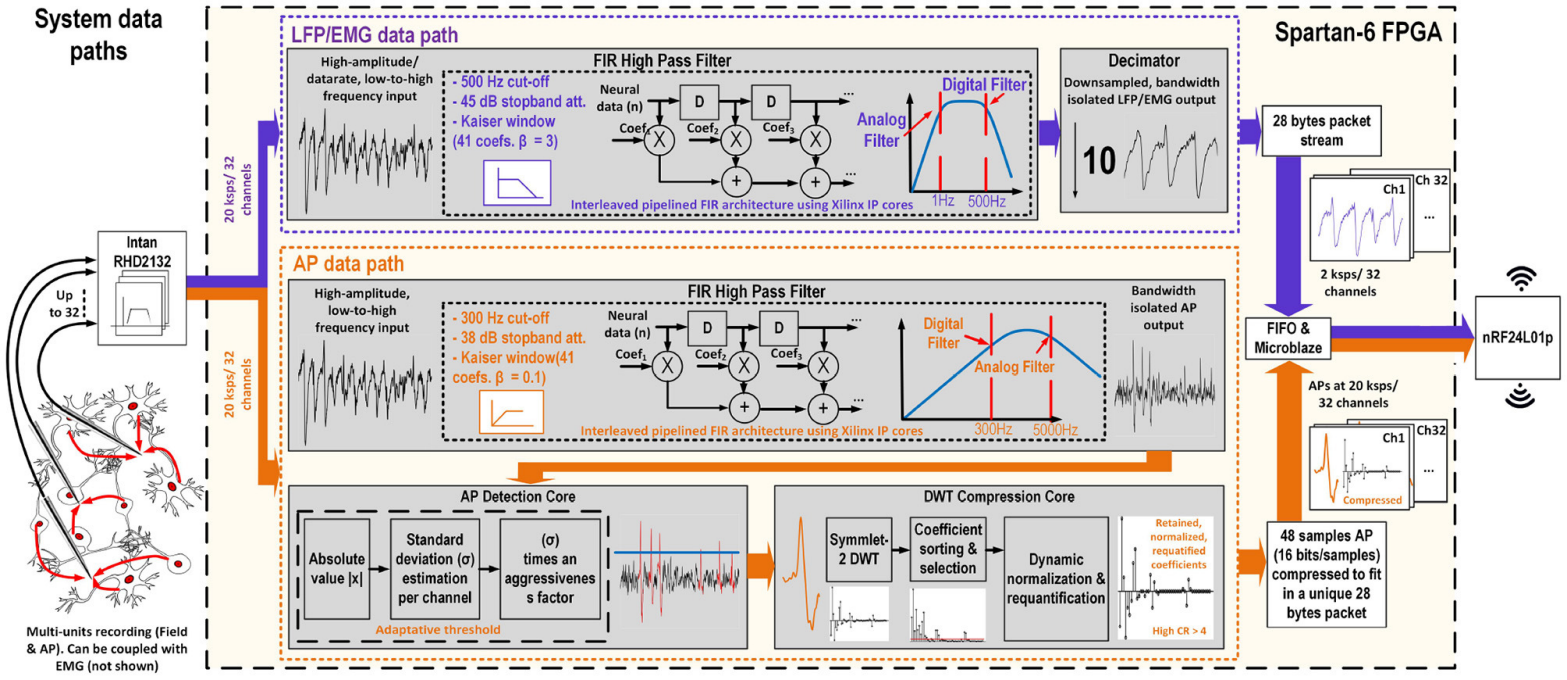
Block diagram of the platform illustrating the data paths for both AP and LFP/EMG signals, and showing the ideal output frequency response for both data paths on the right hand side.

The separation strategy uses both the analog filters inside the RHD2132 neural recording interface and the digital filters implemented inside the FPGA using Xilinx's IP core. Two types of digital filters are utilized: (1) a high pass filter to extract the AP signal and (2) a low pass filter to extract the low frequency signals (LFP/EMG). The same filter design is used both for the LFP and the EMG for preserving the signal integrity, while the sampling rate of the system is set to 20 ksps (4 > 2 × the AP bandwidth) for all signal modalities. Then, the LFP/EMG signals are decimated by a factor of 10 (2 ksps). The data flow for the AP and the LFP/EMG data is illustrated in Figure 3.

First, a tunable analog band-pass filter (0.25–500 Hz 1st order high-pass and 0.1–20 kHz 3rd order Butterworth low-pass) implemented inside the neural recording interface is applied to the neural signal in order to remove any DC offset voltage coming from the electrodes polarization, drift, or high frequency noise and to avoid any aliasing. Then, the FPGA separates the low-frequency from the high-frequency i.e., the AP bands and processes the signals inside two distinct data paths implemented inside the FPGA, as shown in Figure 3. The data paths can be described as follow:

- **High frequency band data path for AP signal processing and extraction**: The raw signal is passed through a finite impulse response (FIR) high-pass filter including 41 symmetrical coefficients to remove any frequencies below 300 Hz. The high-pass filtered AP signal is then sent to the AP detection core. Then, the detected and compressed APs are buffered into a FIFO before being sent to the wireless transceiver over the SPI bus. This path corresponds to the bottom branch in Figure 3.
- **Low-frequency band data path for LFP and EMG signal processing and extraction**: The raw signal is passed through a FIR low-pass filter including 39 symmetrical coefficients to remove any frequency content above 500 Hz. The filtered LFP/EMG signal is then decimated by a factor of 10, to bring its sampling rate down to 2 ksps. The decimated signal is then buffered before being sent to the wireless transceiver. This path is presented in the top branch in Figure 3.

#### 2.2.1. FIR Filters Implementation

The filter designs are optimized to reduce the area utilization inside the FPGA. Since the LFP/EMG signal amplitude is roughly 10 times larger than the AP signal (a little more in the case of the EMG), the AP high pass filter poses an optimization challenge. The stop band attenuation and the filter main lobe width must be optimized to remove any LFP signal at lower frequencies. However, since the FPGA has limited resources and the filtering has to be done in real time, the number of coefficients in the filter is limited, which in turn limits the stop band attenuation and the main lobe width. On the one hand, an optimization must be done to get the maximum number of filter coefficients without generating a delay larger than the sampling frequency. Additionally, the stop band attenuation at low frequencies must be sufficient to remove any other signal modalities, the amplitude of which would be bigger than the APs, so the AP detector can work properly. On the other hand, the main design parameters of the LFP/EMG filter are the *stop band attenuation* and the *number of coefficients*. The signal processing inside the FPGA and the window design must be optimized to meet these design requirements.

Performing all signal processing and system management inside the platform, requires a minimization of the area utilized in order to fit inside the limited FPGA resources, and to reduce power consumption. Therefore, both low and high pass FIR filters, are implemented by reusing the same data path for the 32 channels through a multiplexing scheme. This way, memory and logic blocks utilization is reduced compared to a parallel implementation, which would result in twice the resource used for each additional channel. In addition, the total latency, meaning the time needed to process 32 channels, should be kept lower than one sampling period (1/20*kHz*). Since the FPGA clock (*f*_*CLK*_) is set to 20 MHz, we can estimate the maximum number of clock cycles (*N*_*CYCLES*_) needed for processing one single sample as follows:*N*_*CYCLES*_ = *f*_*CLK*_/(*f*_s_*32). This leads to a maximum processing time of 31.25 clock cycles per sample to avoid any excessive accumulating delay in the filter. To meet this design requirement while maximizing the filter's order, the coefficients in both filters need to be symmetrical. A symmetrical coefficients filter is designed so the first half of the coefficients are repeated in the second half. Having symmetrical coefficients in the filter results in a smaller latency by reusing the multiplication results of the convolution. Since the filter performs a convolution between the filter and the data, we can show that the outcome of each of the multiplication between a sample and a coefficient can be reused when the sample reaches the second appearance of the coefficient in the filter.

In order to meet the attenuation requirements of the filters without exceeding the maximum number of coefficients allowed, windowing optimization was performed. Both filters were designed following the guidelines presented in Rakshit and Ullah (2014). The maximum number of coefficients per window is set to 41 for both filters, so the FPGA module fulfills the latency requirements. However, this can limit the attenuation and the main lobe width of the filters, which are crucial for the AP filter. A Kaiser window was used as suggested in Rakshit and Ullah (2014) for its smaller main lobe width compared to a Chebychev or a Blackman Harris window for an acceptable stop-band attenuation. The Kaiser window, with a β of 0.1 and 41, yielded the highest attenuation in the low frequency band, and has a low ripple in the pass band.

The main parameters of the LFP/EMG filter are the stop band attenuation, and the filter's main lobe width. The Kaiser window also offers a better attenuation in the stop band of a small main lobe width. A Kaiser window including 41 coefficients and a β of 3 was used for this filter.

#### 2.2.2. AP Detection and Compression

In order to achieve 32 neural recording channels, the platform detects the AP waveforms and compresses them. The APs are detected using a digital adaptive threshold and compressed using a four level discrete wavelet transform (DWT) applied on each AP sample. The utilized detection and the compression strategies were previously presented in detail by our group in Gagnon-Turcotte et al. (2019).

### 2.3. Control and Software Management

The platform processes the AP and LFP/EMG data using a dedicated DSP module implemented with custom VHDL modules inside the embedded FPGA. These modules are connected to a *Microblaze* softcore MCU *via* an AXI-Lite bus. The system can handle the high level functions, like the communication with the wireless transceiver and the commands interpretation, using a firmware written in C code and running inside the *Microblaze*, while the VHDL custom modules sends their critical notifications through the interrupt controller. The firmware triggers an interrupt when a detected/compressed AP sample or a LFP/EMG sample is ready to be sent to the transceiver.

Each compressed AP (48 samples on 6-bits) is inserted within a single 28 bytes packet, which also includes the type of packet code (8 bits AP packet code), the AP detection timestamp (24 bits), and the channel number (5 bits). Similarly, the LFP/EMG data are packetized and sent within separate packets, each of which includes the packet type (LFP/EMG) and the channel number of the first sample of the packet.

On the base station side, the LFP/EMG data is routed directly to the user interface for plotting and/or storing the data, while the AP data is routed to the decompression module for waveforms reconstruction before the reconstructed signals can be sent to the user interface. The reconstructed AP and LFP/EMG signals are displayed separately inside the user interface using distinct time and amplitude scales.

### 2.4. Experimental Setups for the *in vivo* Experiments

#### 2.4.1. Multimodal Electrophysiology Recordings

**Figure 4:**
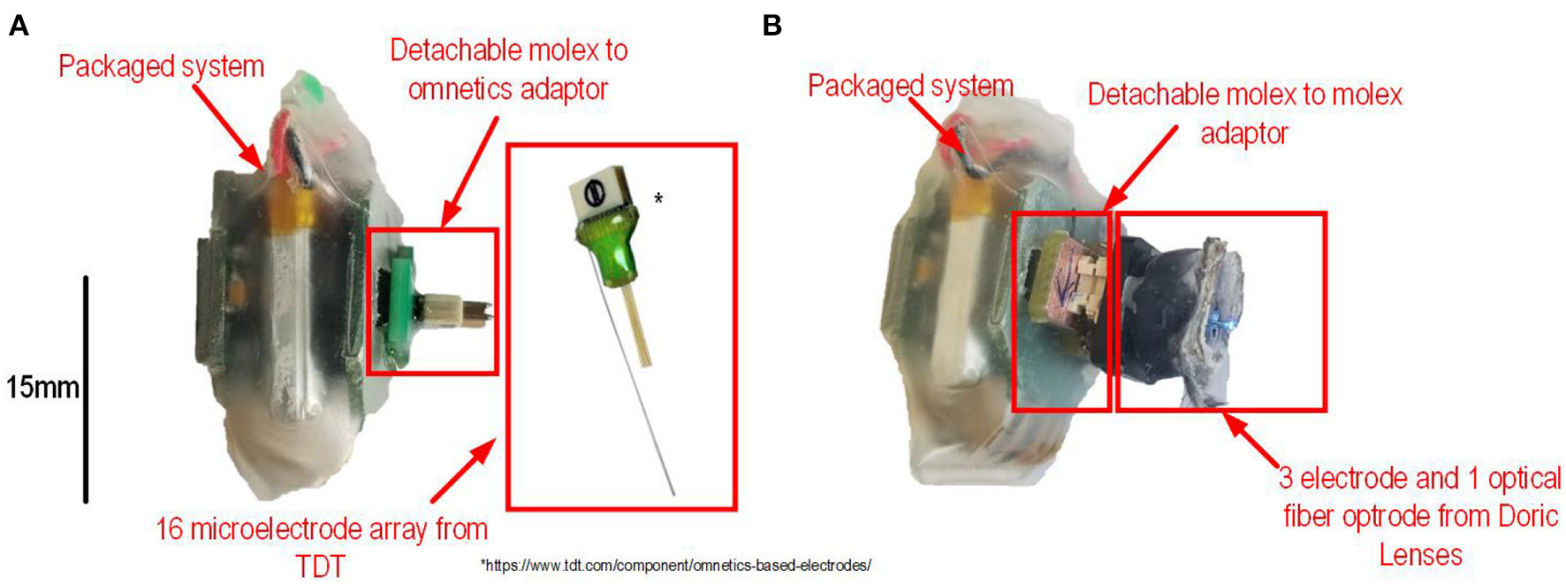
**(A)** Packaged system connected to a Molex to Omnetics adaptor, a 16 channels microelectrode array and a ground wire. **(B)** Packaged system connected to a Molex to Molex adaptor and a three electrodes and 1 optical fiber optrode from *Doric Lenses*.

The encapsulated system is used with an adaptor to interface with a 16-microelectrode implant. This adaptor allows to interface a *Molex* connector (on the platform) with an *Omnetics* A79042-001 including 16 pins (on the microelectrode implant). The implant consists of a 16 electrode *Omnetics* based microwire array from *Tucker-Davis Technologies Inc*. (TDT, 2021). The electrodes are separated in two rows of eight electrodes. Electrodes within rows are separated by 250 μm, and rows were 500 μm apart. Each microwire electrode, the diameter of which is 33 μm, is made from polyimide-insulated tungsten. The implant has one reference wire and uses an *Omnetics* A79043-001 connector to interface with the adaptor. Figure 4A shows all the components of the platform before implantation.

All animal manipulations were performed with the approval of Université Laval's Animal Protection Committee (CPAUL) Protocol # VRR-2019-007. A Long-Evans rats (300 g female) was implanted with a 16-channel electrode array made of a 2 × 8 assembly of 33 μm tungsten microwires (Tucker-Davis Technologies Inc.) to acquire electrophysiological data *in vivo*. The tips of the microelectrodes were inserted ~1.25 mm deep into the left primary motor cortex (centered at 1 and 3 mm anterior and lateral with respect to bregma). A first set of data was recorded peri-operatively during Ketamine-Xylazine anesthesia (10–100 mg/kg). A second set of recordings was performed 7 days later during live behavior with the same rat.

#### 2.4.2. Optogenetic Stimulation and AP Recording

The extracellular signals were recorded with a Fi-Wi opto-electric cannula (3x tungsten 25 μm probes; 1x optical fiber 245 μm 0.37 NA; *Doric Lenses*, Canada) connected to the platform. The setup, which is using a Molex to Molex adaptor and the optrode, is shown in Figure 4B. The Thy1::ChR2-EYFP mouse was purchased from the Jackson Labs [B6.Cg-Tg(Thy1::COP4/EYFP)18Gfng/J]. A small craniotomy was made over the CA1 of dorsal hippocampus (rostro-caudal: −2.5, medial-lateral: 2.5, from Bregma) while the mouse was maintained on isoflurane anesthesia. The cannula was lowered above the CA1 pyramidal cells (dorso-ventral: −1.75), 500 ms pulses of light were delivered with a delay of 4.5 s between the pulses while the raw electrophysiological signal was recorded at a sampling rate of 20 ksps.

#### 2.4.3. EMG Responses to Optogenetic Stimulation of the Motor Cotex

A Long-Evans rat was used to conduct an optogenetic stimulation experiment in the motor cortex. Channelrhodopsin-2 was virally expressed in the rat's motor cortical pyramidal neurons following an injection of the AAV2/php.eB-CaMKIIa-hChR2(H134R)-mCherry viral construct, developed at the Canadian Neurophotonics Platform Viral Vector Core Facility (RRID:SCR_016477). In brief, the AAV was prepared in the absence of helper virus. It was purified on an iodixanol gradient from cell culture. The virus was resuspended in PBS 320 mM NaCl + 5% Sorbitol + 0.001% Pluronic F-68. This rat was implanted with a 400-μm optical fiber, tapered over 1.5 mm, to illuminate the infected cortical tissue in the primary motor cortex. Additionally, three pairs of multi-stranded, PFA-coated stainless steel microwires (41AWG, A-M Systems Inc.) were inserted intramuscularly in the right trapezius, extensor carpi radialis and flexor carpi radialis muscles, and tunneled subcutaneously to a connector (Samtec Inc.) secured on the rat's skull using dental acrylic. A custom-made adapter was made to perform simultaneous optogenetic cortical stimulation and EMG recordings using the wireless platform. During this experiment, EMG signals were recorded and transmitted directly to the base station using the wireless platform, which also provided a control signal to drive the activation of an external light source (488 nm laser diode, Doric Lenses Inc.), to which the animal was tethered using an optical patch cord.

## 3. Results

### 3.1. Measured Performance and Results

#### 3.1.1. Filters Responses

**Figure 5:**
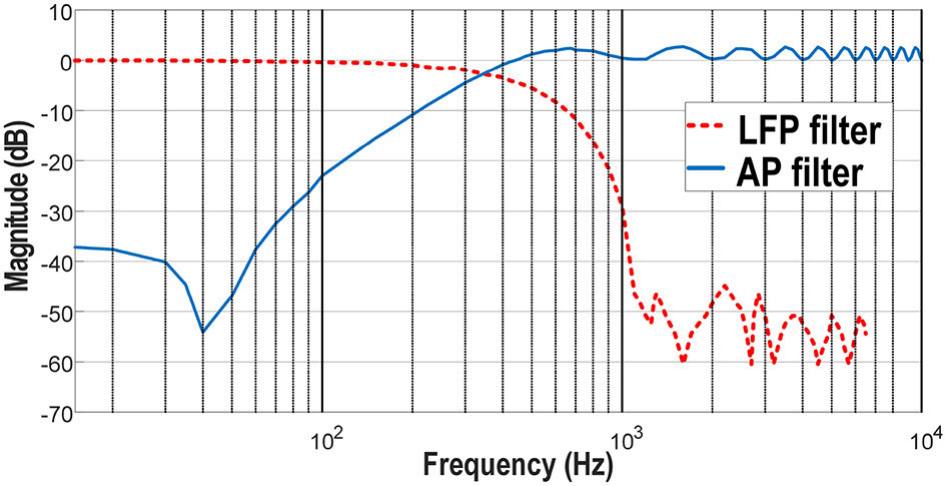
In blue/plain, frequency response of the digital AP high-pass filter. In red/dotted, frequency response of the digital LFP low-pass filter.

The measured frequency responses of the AP filter and the LFP/EMG filter are shown in Figure 5. The responses address the design requirements i.e., 500 Hz low pass cut frequency for the LFP filter with −50 dB attenuation in the stop band, and 300 Hz high pass cut frequency with −40 dB attenuation in the stop band. A summary of these characteristics is presented in Table 1.

**Table 1:** Platform's characteristics and performance.

**Parameter**	**Value**
Nb of recording/stimulation channels	32/4
Targeted signals	AP/LFP/EMG
ADC resolution	16 bits
Sampling rate	20 ksps
Analog low-pass filter cut-off freq.	0.1–20 kHz
Analog high-pass filter cut-off freq.	0.1–500 Hz
Digital low-pass filter cut-off freq.	500 Hz
Digital low-pass filter stop-band att.	45 dB
Digital high-pass filter cut-off freq.	300 Hz
Digital high-pass filter stop-band att.	38 dB
Digital filters coefficient number	40
Combined data reduction ratio^*^	7.77
Dimensions(LxDxH) packaged	2.8 × 1.5 × 1.1 cm^3^
Maximum current per stim. channel	200 mA
Power consumption(w/o stim.)	37 mA
Weight(w/ and w/o battery)	4.4/1.7 g

* *AP firing rate of 50 AP/s*.

#### 3.1.2. Power Consumption

The system's total current consumption without the filtering core and the dual data path was measured at 36 mA while recording AP and LFP signals that were recorded in the brain of a mouse in the course of previous *in vivo* experiments that were played at the inputs of the platform using a *Tektronix AFG 3101* arbitrary waveform generator over 32 channels. The digital filtering core and additional digital signal processing cores, which are critical to enable multimodal electrophysiological recordings in this application, was found to add only 1 mA (1.2 mW in the FPGA core powered at 1.2-V) while the system is recording from 32 channels over both signal frequency bands. The measured recording autonomy with the dual data path activated over 32 channels in parallel and without light stimulation was 94 min, using a 100-mAh battery.

#### 3.1.3. System Performance

The *Combined Data Reduction Ratio* (CDRR) metric presented in Table 1 was evaluated using a typical firing rate of 50 AP/s, while recording both AP and LFP separately at 20 ksps on 16 bits. This ratio was calculated as follows:

(1) $$\begin{array}{r} CDRR=\frac{f_{SH}}{\frac{NFR\times Samples_{AP}}{CR}+f_{SL}} \end{array}$$

where *f*_*SH*_ is the sampling frequency of the raw signal in ksps, NFR is the neuron firing rate, *Samples*_*AP*_ is the number of samples in each AP, CR is the compression ratio for the AP (CR) and *f*_*SL*_ is the decimated sampling frequency in the low frequency band. With a *f*_*SH*_ of 20 ksps, a NFR of 50 AP/s, a *Samples*_*AP*_ of 48 Samples/AP, a CR of 4.17 and a *f*_*SL*_ of 2 ksps, the CDRR was 7.77. This metric takes into account the data reduction induced both by the AP detection core and by the wavelet compression core. These characteristics of the system are summarized in Table 1.

#### 3.1.4. Synthetic Recording Results

**Figure 6:**
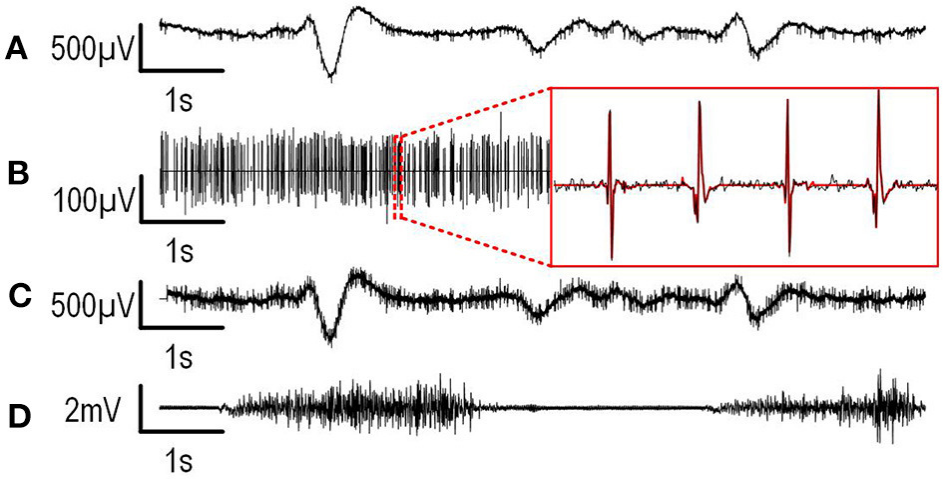
Recordings of synthetic signals are presented. **(A)** Digitally filtered and decimated LFP signal. **(B)** Recorded, digitally filtered, detected, and compressed APs. The magnification shows the uncompressed AP signal (black) superimposed with the decompressed detected APs (red). **(C)** Recorded original raw signal. **(D)** EMG recorded in parallel with the neural signal.

To validate whether the system can effectively record and separate AP, LFP, and EMG signals simultaneously in real time, the EMG, AP and LFP signals recorded in the brain of a mouse in the course of previous *in vivo* experiments were played at the inputs of the platform using a *Tektronix AFG 3101* arbitrary waveform generator. The signal was attenuated using a precise voltage divider, and then applied on 31 recording channels simultaneously, while the last channel (channel 32) was connected to the pre-recorded EMG signal. The EMG signal was recorded on the arm of a human volunteer using Ag/AgCl surface electrodes and then passed through a 60 Hz notch filter in post processing. Both the real EMG signal and synthetic neural signal references were connected to the ground of the system and on the Intan RHD2132 input reference pin. Figure 6 presents the recorded data on one channel of both LFP and AP and on the EMG channel. Figures 6A,B show the results for the digitally separated signal shown in Figure 6C. This former subfigure shows that both the AP and LFP signals were properly extracted, which allows to correctly detect and compress the APs during subsequent steps. Figure 6D shows the EMG data that was recorded in parallel on channel 32.

### 3.2. *In vivo* Results

Four separate experiments were conducted to validate the critical functionalities of the presented system *in vivo* and with freely moving rodents. First, an electrophysiological experiment was performed *in vivo* in an anesthetized rat with a 16-microelectrode array implanted in the primary motor cortex to demonstrate the real-time separation of the APs and LFPs over a high channel count. Secondly, electrophysiology recordings were performed in freely moving conditions with the same implanted rat. Third, EMG recordings and optogenetic stimulation of the motor cortex were performed *in vivo* in a Long-Evans rat expressing ChR2 delivered with a viral construct. In this experiment, three pairs of electrodes were inserted intramuscularly in the right trapezius, extensor carpi radialis and flexor carpi radialis muscles and paired with optogenetic stimulation performed in the primary motor cortex. Finally, the system was used to perform optogenetic stimulation in the brain of a transgenic Thy1::ChR2-EYFP mouse implanted with an optrode manufactured by Doric Lenses, Canada including three recording channels and one stimulation channel. The methods of these *in vivo* tests and their experimental results are reported in the following sections.

#### 3.2.1. Multimodal Electrophysiology Recording Results

To validate the platform ability to properly collect and separate LFP and AP signals during a freely moving experiment, electrophysiological recordings were conducted *in vivo* in an anesthetized and a freely moving rat using a 16 micro-electrode array recording both LFP and AP signals in parallel. AP were detected and compressed using wavelet compression by the system on 16 channels in real time. The LFP signals were decimated by the DSP core that is implemented inside the FPGA. The DSP core could successfully reduce the output data rate of the platform below the maximum data rate which can handle the wireless transceiver. Indeed, we can show that the minimum data rate needed to record the full signal bandwidth over 16 channels in parallel at 20 ksps, without data reduction, is 5.12 Mbps. Our data reduction strategy implemented in the platform reduces the data rate below the maximum effective data rate of the wireless transceiver, which is limited to ~1.4 Mbps.

#### 3.2.2. Anesthetized Multimodal Recording Results

The data presented in this section comes from recordings that were performed during the implantation procedure described in section 2.4.1, while the rat was anesthetized and remained still. These recordings were taken after the implantation and before solidifying the implant for chronic use. The platform recorded AP and LFP signals on 16 electrodes during this experiment. The APs that were detected and compressed by the platform were wirelessly transmitted to the base station during the experiment.

**Figure 7:**
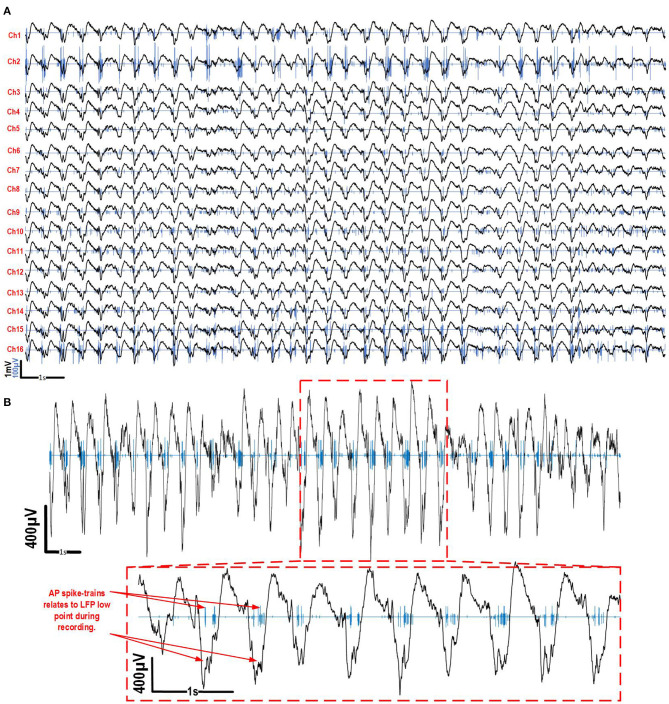
*In vivo* recordings for 16 channels of both LFP and AP in the brain of a freely moving Long-Evans rat under anesthesia. **(A)** 20 s of recording during the experiment with the AP and LFP superimposed. **(B)** A magnification of the signals on channel two during the same recording to highlight the correspondence between AP spike-trains and LFP activity.

In order to analyze the AP and LFP signals recorded on the 16 microelectrodes, the AP trains were reconstructed by temporally placing the AP samples according to their timestamps with respect to their detection time point. They were then superimposed with the decimated LFP signals (Figure 7A). As expected, under ketamine-xylazine anesthesia cortical activity revealed slow oscillation pattern (major frequency about 1 Hz). The recorded neurons were silent during the depth-positive components and they fired AP during the depth-negative components of the slow oscillations. For channels 2, 14, 15, and 16 large APs (up to 250 μV peak to peak) were detected in separated rapid spike-trains which is expected under ketamine-xylazine anesthesia in the neo-cortex. In the rest of the channels, smaller APs (<100 μV peak to peak) and spiking activity was recorded. Among the LFP shown in Figure 7A, a large low-frequency component can be seen over the 16 channels. Figure 7B shows AP and LFP signals superimposed over 20 s of a 30 s recording on electrode 2, with a zoom on a section of the signal, to show the relation between AP firing and phase of LFP. This correspondence between both measured modalities show the correct parallel recording capability of the presented platform when utilized into an *in vivo* setting.

**Figure 8:**
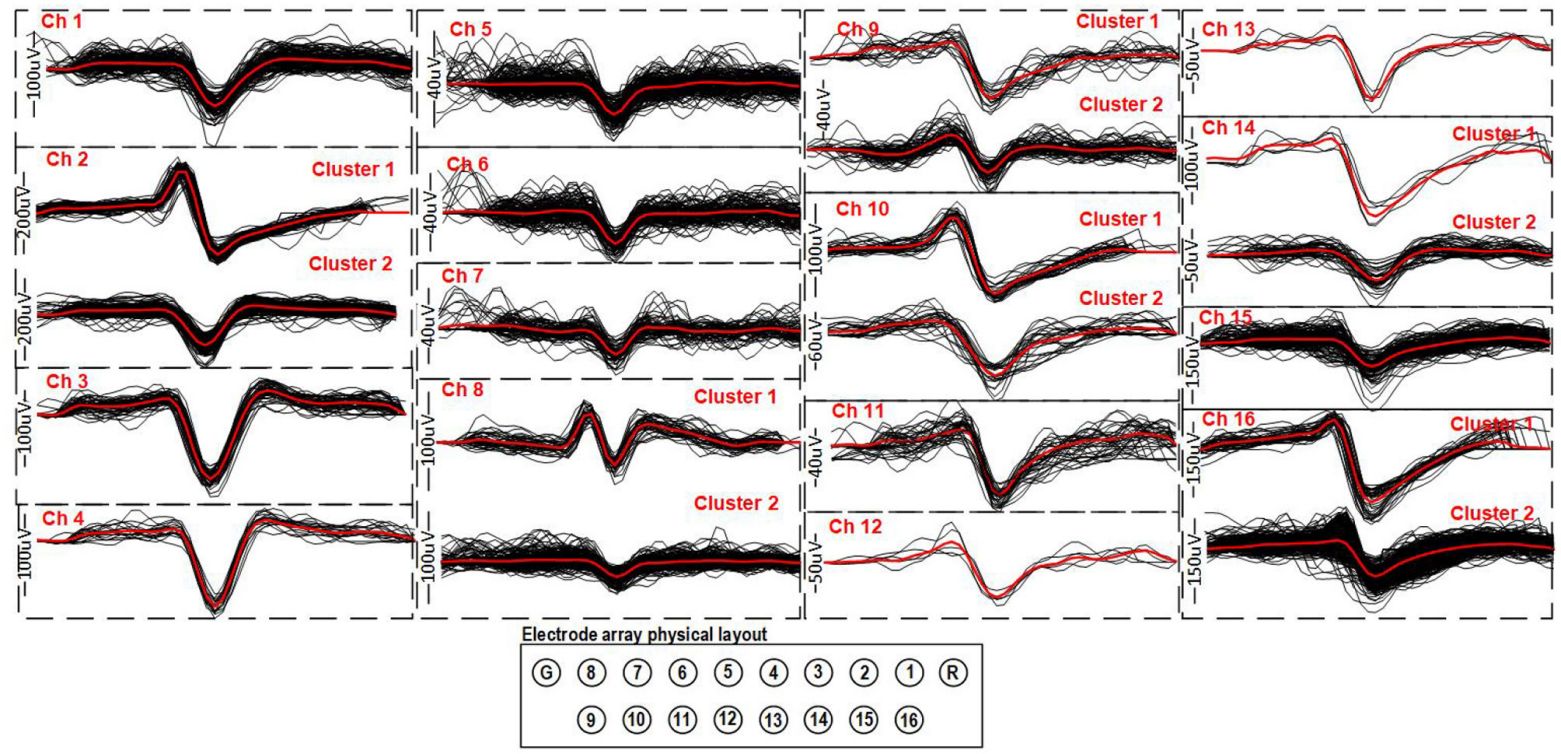
Clustered APs detected for 16 channels recorded simultaneously during a 30 s period. The average AP for each cluster is displayed in red in order to better visualize its shape. Each AP waveform is 2.4 ms.

The APs that were detected during 30 s over 16 channels were clustered and superimposed (Figure 8). The decompressed APs recorded during the experiments were re-filtered from 300 Hz to 5 kHz and re-centered around their peak value in post-processing. A Principal component analysis (PCA) has been performed on the resulting extracted APs for each channel and followed by a clustering task using the Kmeans algorithm to identify and sort the different AP shapes collected over each electrode, which result from different neurons. While channels 5, 6, and 7 showed small waveforms (<20μV), most channels showed at least one AP shape that is repeated throughout the recording, indicating that the electrodes were effectively picking up genuine neural activity for these channels. This shows that the AP detection and compression strategy implemented inside the platform and utilized to perform this experiment, works properly along with our signal separation method to capture different signal modalities in parallel over several channels.

The data reduction on the platform during recording was calculated using a 30 s sample of the recording session. The total number of APs detected during 30 s over the 16 channels was 5,547 (Figure 7A). Given that each AP includes 48 samples on 16 bits per sample, the decimated sampling frequency of the LFP is 2 ksps and the compression ratio is 4.17. The total data reduction ratio can be calculated as follows:

(2) $$\begin{array}{r} CDRR=\frac{20ksps\times 30s\times 16}{\frac{5547APs\times 48samples/AP}{4.17}+2ksps\times 30s\times 16} \end{array}$$

The resulting total data reduction ratio for this experiment was 9.38, which reduces the data rate from 5.12 Mbps, for full bandwidth neural signals, to 0.55 Mbps, which is well below the limits of our low-power wireless transceiver chip after data reduction.

**Figure 9:**
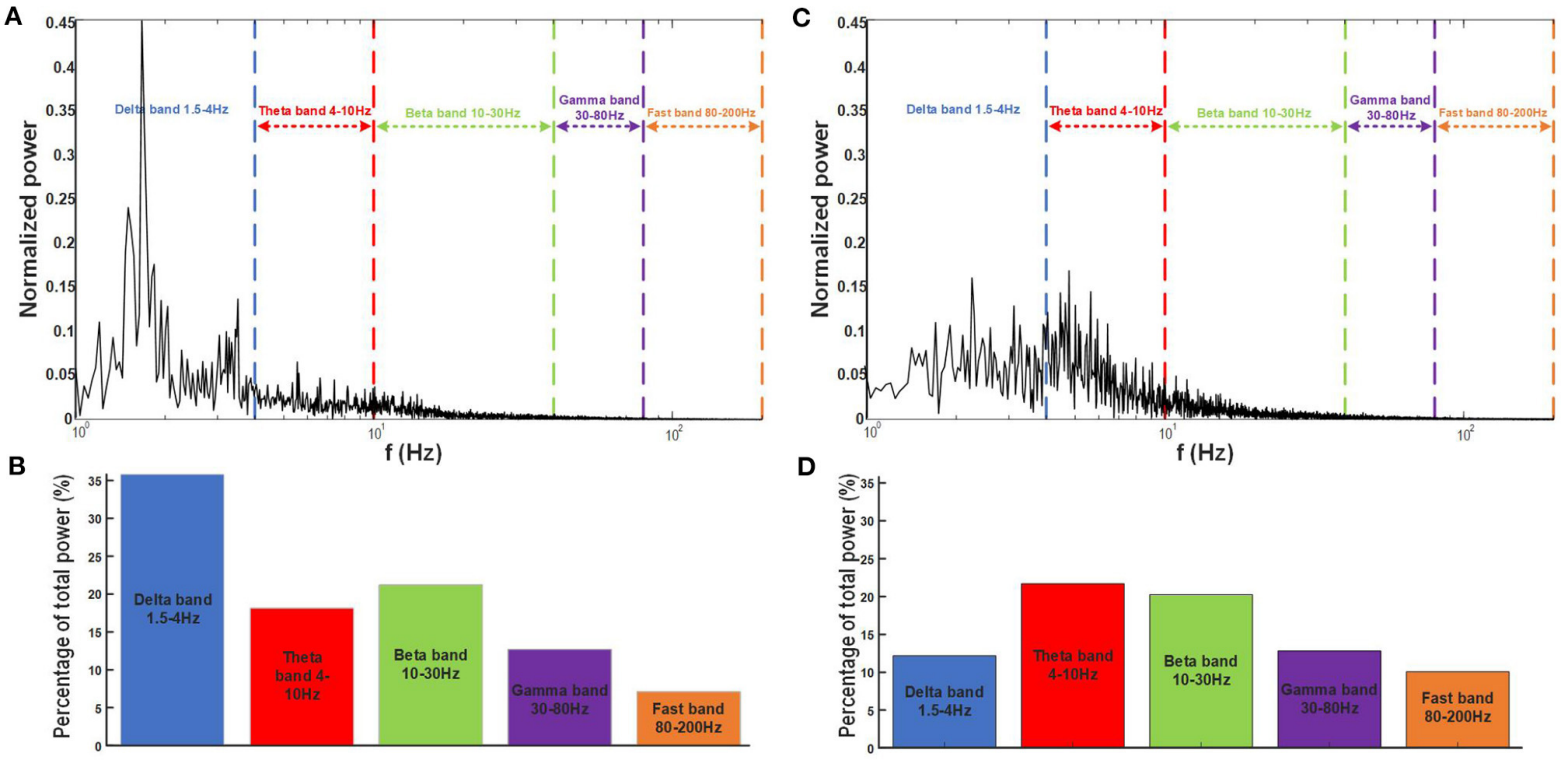
**(A)** Frequency content for a 30 s LFP recording on one electrode of the 16 electrode implant in the primary motor cotex of the Long-Evans rat under anesthesia with the traditional frequency bands used for analysis identified on the graph. **(B)** Power percentage contained in each band for the specific recording in anesthesia. **(C)** Frequency content for a 20 s LFP recording on the same electrode as in **(A)**, a week after implantation while the rat is freely moving with the traditional frequency bands used for analysis identified on the graph. **(D)** Power percentage contained in each band for the specific recording for the freely moving experiment.

In order to obtain the frequency content of the LFPs during the recording session, a fast Fourier transform (FFT) was performed on the LFP recorded on channel 2 (Figure 9A). The result of the FFT was then normalized using the 2-norm method. The typical frequency bands used in the LFP analysis (Liu et al., 2017) are added to Figure 9A as an indicator to better analyze the spectrum presented here. Figure 9B shows the power distribution in each frequency band. The majority of the activity is contained in the two frequency bands below 10 Hz, which is typical with Ketamine-Xylazine anesthesia in the neocortex.

#### 3.2.3. Freely Moving Results

The results presented in this section show the device's ability to record multimodal electrophysiology signals simultaneously in a freely moving laboratory animal. Recordings were taken 1 week after implantation in the same Long-Evans rat that was used in section 2.4.1. AP and LFP signals were simultaneously recorded from the 16 electrodes using the method described in section 3.2.2, while the animal was freely behaving. The packaged system weight 4.68 g, which represents only 1% of the 300 g Long Evans rat.

**Figure 10:**
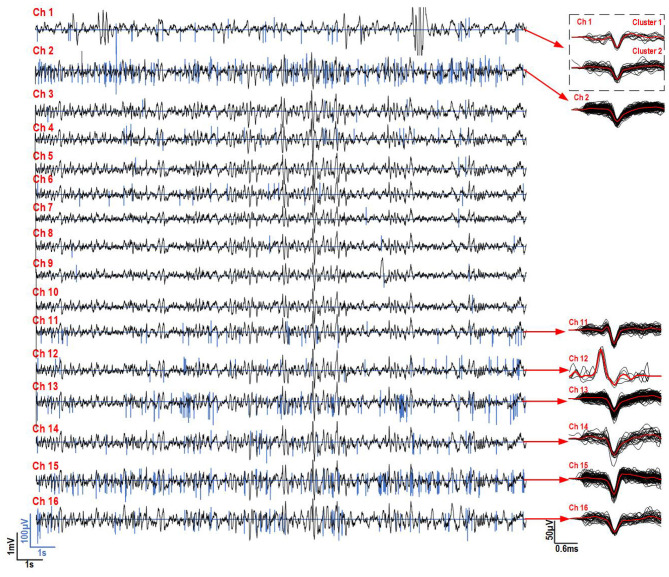
*In vivo* recordings for 16 channels of both LFP and AP in the brain of a freely moving Long-Evans rat with the implant and system on its head. On the left, 20 s of recording during the experiment with the AP and LFP superimposed is shown. On the right, the clustered and superimposed APs are shown along with the average AP waveform (in red) for the channels were some activity was detected is shown.

As in section 3.2.2, the AP and LFP signals recorded on the 16 microelectrodes were reconstructed by temporally replacing the AP samples according to their timestamps with respect to their detection time point and were superimposed with the decimated LFP signals (Figure 10). Figure 10 shows the superimposed results of both signals collected during a 20 s part of the recording. The APs detected on their respective channels were sorted and clustered using a PCA and the Kmeans algorithm to identify and sort the different AP shapes collected over each electrode, which result from different neurons. The result is shown on the right hand side of Figure 10. An AP shape was detected consistently during the experiment for channels 1, 2, and 11–16. Little to no activity was detected on the other channels.

In order to compare the frequency content of the LFP during exploration with the frequency content during anesthesia (section 3.2.2), an FFT and 2-norm normalization were performed on the LFP recorded on channel 2 during the freely moving experiment (Figure 9C). The power distribution was calculated comparing the total power with the power in each power bands identified in Figure 9D. These results show a shift between the frequency content measured during the anesthesia (Figures 9A,B) and the beginning of the freely moving experiment (Figures 9C,D), as expected, during exploration LFP signal was dominated by activities in theta and beta frequency range.

### 3.3. Optogenetic Stimulation and AP Recording Results

**Figure 11:**
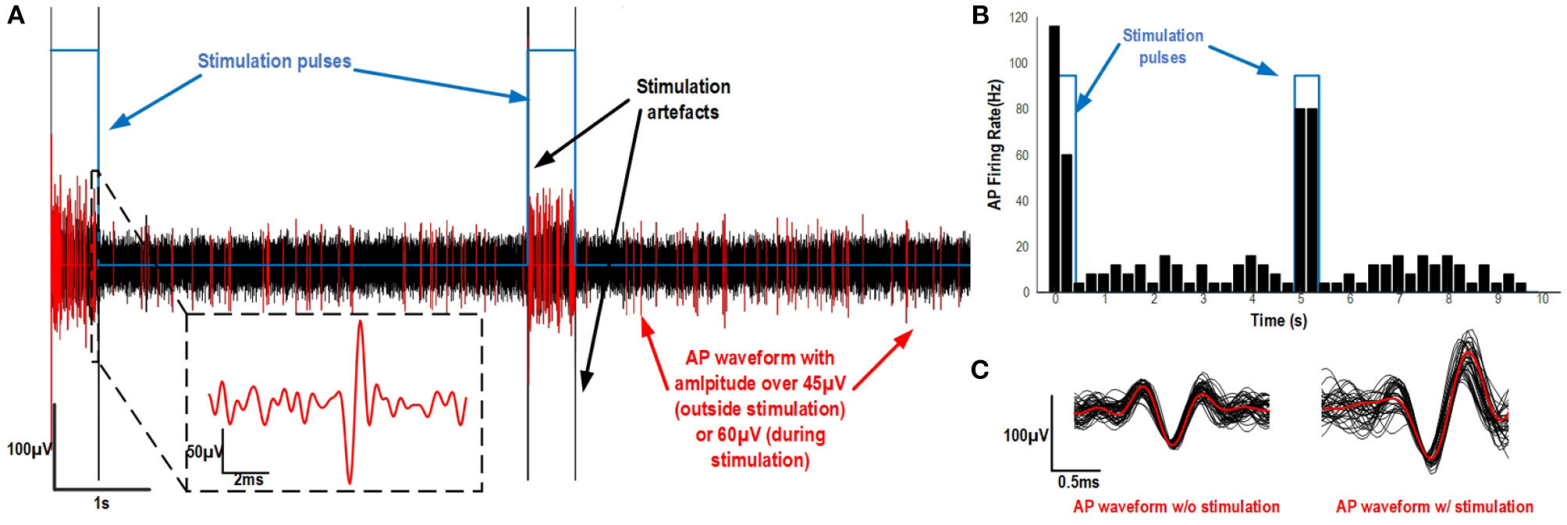
**(A)** Recorded signal from one electrode of the optrode implanted in the Thy1::ChR2-EYFP transgenic mouse. Five hundred milliseconds of stimulation pulses were applied with a 4.5 s delay between each pulse. The simulation pattern is shown in blue, the recording in black and the detected AP waveforms in red. A zoom on an action potential of about 200 μV is added to better visualize the activity present during the light pulses. **(B)** The AP firing rate at each moment of the experiment. **(C)** The clustered AP waveforms w/o stimulation and during the stimulation pulses.

The extracellular activity of CA1 pyramidal neurons was recorded with the system in an anesthetized Thy1::ChR2-EYFP mouse with the setup described in section 2.4.2. The recorded signal was filtered from 300 Hz to 3 kHz for the three recording electrodes. A fixed threshold of 60 μV (inside stimulation pulses) and 45 μV (outside stimulation pulses) was applied after the experiment to better identify the events during the recording and highlight the increase in activity during the optogenetic stimulation shown in blue (Figure 11A). The firing rate during the experiment was also calculated for each 0.25 s segment of the recording (Figure 11B) and revealed a relation between stimulation and an increase in AP firing rate for the experiment. The APs detected during and in between stimulation were sorted and clustered separately using a PCA and the Kmeans algorithm (Figure 11C). The AP shapes identified were higher in amplitude during stimulation as compared to the APs in between the stimulation pulses. Overall, delivering 500 ms light pulses resulted in an increased firing rate and higher AP amplitude during the pulses. The optical stimulation also introduced small stimulation artifacts (likely due to photoelectric effect, shown in Figure 11A) in the recorded signal when turning the LED on and off.

### 3.4. Results of EMG Responses to Optogenetic Stimulation of Motor Cortex

In order to demonstrate the capability of the system to record EMG and to control a stimulation light source, EMG were recorded using the setup described in section 2.4.3. This setup served as a proof-of-concept experiment mainly for the EMG recording, as the platform's capability to generate light for optogenetic stimulation was already demonstrated in section 3.3.

**Figure 12:**
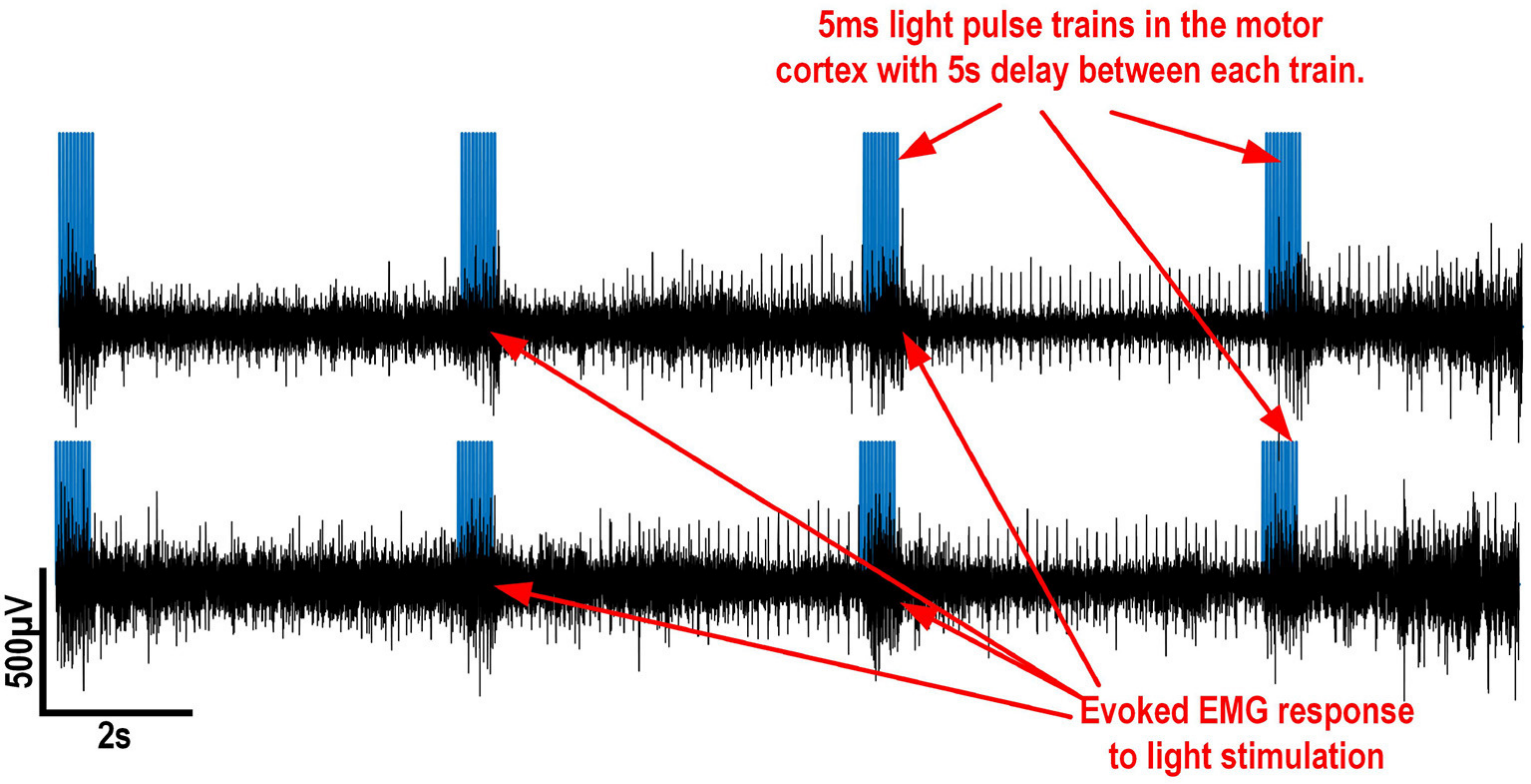
*In vivo* EMG recording in the extensor carpi radialis and flexor carpi radialis muscles of a freely moving rat. The stimulation trains are shown in blue to show when a response should be evoked.

During the experiment, a stimulation train of 10 pulses of 5 ms each 50 ms is repeated every 5.5 s to the optical fiber for 20 s in order to generate sustained activity during every stimulation train. Since the platform measures the signal between 2 wire electrodes and a common reference, which is located far from each muscle, signals are subtracted together to reduce the background noise from the nearby muscle fibers. Figure 12 shows EMG signals recorded during the experiment. The muscle's EMG activity level increases during the optogenetic stimulation pulse-trains.

## 4. Discussion

We presented the design of a wireless electro-optic platform and its utilization to perform multimodal neural recording and optical stimulation in parallel with freely moving rodents. The system allows the digital separation of AP and LFP/EMG signals in real time, provides AP detection and AP wavelet compression to minimize the data rate of the wireless link and the overall power consumption. This unique feature, combined with the decimation of the LFP, allows to significantly reduce the amount of data to be transmitted over the low-power wireless data link, while preserving the integrity of the extracted signal modalities. The platform was thoroughly measured and validated in the Smart Biomedical Microsystems Lab and at the CERVO Brain Research center during *in vivo* neurophysiological experiments. Electrophysiology recordings were performed during both anesthetized and freely moving conditions using a 16-channel microelectrode array implanted in the primary motor cortex of a rat. AP and LFP were recorded in parallel using the platform on 16 channels, thanks to the real-time signal separation, AP dectection/compression and LFP decimation, during a behavioral experiment. Optogenetic stimulation along with electrophysiology recordings were performed in parallel in the brain of a transgenic mouse using an optrode including 3 electrodes and 1 optical fiber. EMG recordings along with optical stimulation were also performed respectively in the arm and in the motor cortex of a rat expressing ChR2 *via* a viral vector. These three experiments allowed to validate the platform thoroughly in many experimental scenarios. In future work, we intend to use the presented platform inside a real-time closed-loop scheme where electrophysiological recordings obtained from one specific area will be used to trigger and control optical stimulation into other areas of the brain.

## Data Availability Statement

The raw data supporting the conclusions of this article will be made available by the authors, without undue reservation.

## Ethics Statement

The animal study was reviewed and approved by Université Laval's Animal Protection Committee (CPAUL).

## Author Contributions

GB designed the multimodal extraction strategy and the low-frequency data path. He measured the performance of the system, participated in the *in vivo* validation, analyzed the results, and he wrote this manuscript. GG-T designed the system and the high-frequency data path. He participated in the *in vivo* validation and in the writing of this paper. LG participated in the design of the system. IK participated in the *in vivo* validation and in the writing. YD, IT, and CE supervised the *in vivo* validation and participated in the writing of the paper. BG supervised and participated in the design of the system and participated in the writing of the paper. All authors contributed to the article and approved the submitted version.

## Conflict of Interest

The authors declare that the research was conducted in the absence of any commercial or financial relationships that could be construed as a potential conflict of interest.

## Publisher's Note

All claims expressed in this article are solely those of the authors and do not necessarily represent those of their affiliated organizations, or those of the publisher, the editors and the reviewers. Any product that may be evaluated in this article, or claim that may be made by its manufacturer, is not guaranteed or endorsed by the publisher.
